# A RPCA-Based ISAR Imaging Method for Micromotion Targets

**DOI:** 10.3390/s20102989

**Published:** 2020-05-25

**Authors:** Liangyou Lu, Peng Chen, Lenan Wu

**Affiliations:** 1 School of Information Science and Engineering, Southeast University, Nanjing 210096, China; luliangxuexi@126.com (L.L.); wuln@seu.edu.cn (L.W.); 2 State Key Laboratory of Millimeter Waves, Southeast University, Nanjing 210096, China

**Keywords:** ADMM, ISAR, micro-Doppler, RPCA

## Abstract

Micro-Doppler generated by the micromotion of a target contaminates the inverse synthetic aperture radar (ISAR) image heavily. To acquire a clear ISAR image, removing the Micro-Doppler is an indispensable task. By exploiting the sparsity of the ISAR image and the low-rank of Micro-Doppler signal in the Range-Doppler (RD) domain, a novel Micro-Doppler removal method based on the robust principal component analysis (RPCA) framework is proposed. We formulate the model of sparse ISAR imaging for micromotion target in the framework of RPCA. Then, the imaging problem is decomposed into iterations between the sub-problem of sparse imaging and Micro-Doppler extraction. The alternative direction method of multipliers (ADMM) approach is utilized to seek for the solution of each sub-problem. Furthermore, to improve the computational efficiency and numerical robustness in the Micro-Doppler extraction, an SVD-free method is presented to further lessen the calculative burden. Experimental results with simulated data validate the effectiveness of the proposed method.

## 1. Introduction

Inverse synthetic aperture radar (ISAR) can provide two-dimensional (2D) high-resolution images of non-corporative moving targets, and it plays an important role in military and civil applications such as automatic target recognition (ATR) and target classification [1,2,3]. The conventional ISAR imaging system achieves high range resolution by emitting wideband waveforms, while the high cross-range resolution is acquired by a large aspect angle variation of the target with respect to the line of sight (LOS) [1]. Motions between radar and target include translational motion along LOS and rotation around the equivalent imaging center. As the translation brings about range profile misalignment and phase defocusing leading to failing in imaging, the processing of translation compensation must be performed in advance to preserve the effective rotational motion of the target before azimuth compression for ISAR imaging. Supposing the translation compensation has been effectively accomplished, satisfactory focused imagery with higher cross-range resolution can be achieved by using range-doppler algorithm (RDA) under the assumption of uniform rotation in longer correlation processing time (CPI) [4]. However, the assumption may hardly be satisfied with some practical applications. When the radar works in multi-function mode, the echo data collected from short CPI or discontinuously sparse aperture (SA) is often limited or incomplete. In these cases, RDA and the methods based on modern spectrum estimation [5,6] fail to provide clear images of the target because of the high-level sidelobes resulting from zero padding, data interpolation and/or model mismatch.

To overcome the drawbacks of these methods, compressive sensing (CS) [7,8,9] technique has been introduced in high-resolution ISAR imaging, and plenty of works were reported recently [10,11] where the basic idea behind these works is to formulate ISAR imaging as a sparse signal recovery problem. The CS theory states that a signal having a sparse representation can be recovered exactly from a small set of linear, non-adaptive measurements. For the ISAR image, the dominant scattering centers of a target occupy only a few cells in the imaging plane, which exhibits a sparse feature that paves the way to apply CS to achieve high-resolution ISAR imagery.

It is often that the target or some structures on it are vibrating or rotating aside from the bulk motion. These vibrations or rotations are referred to as micromotion dynamics [12]. Additionally, the micromotion dynamics also exist in nonrigid targets, such as the rotating propeller of a fixed-wing aircraft, the rotating rotor blades of a helicopter, a rotating antenna, etc. Target micromotion introduces additional time-varying frequency modulations on the radar echo and generates sidebands about the Doppler frequency induced by the main body, which is known as the Micro-Doppler effect [12,13] (also called the Micro-Doppler interference or micromotion signal or Micro-Doppler signal). In such a situation, the ISAR image of the main body is usually contaminated, particularly when the Micro-Doppler interference is emphatic. Consequently, Micro-Doppler extraction and separation must be properly conducted in ISAR imaging for the target with micromotion parts to acquire a clear image of the main body, and there has been increasing attention in this study in recent years.

For micromotion scatterer with a large rotating radius, it generates a Micro-Doppler signal, which exhibits sinusoidal modulation in the spectrogram [14,15] after range compression, whereas the doppler signal from the main body scatterer shows the shape of straight lines. Based on the difference of shape in the spectrogram, some approaches have been proposed to eliminate the Micro-Doppler signal. In [13], Li and Ling proposed an adaptive chirplet decomposition algorithm to extract the Micro-Doppler signal by chirp-rate thresholding with high computation burden. Zhang et al. extracted the Micro-Doppler signal by using the Hough transform in the spectrogram, which its performance heavily depends on the quality of the ISAR image [16]. In [17], a spectrogram cancellation method was employed to implement the separation of the Micro-Doppler signal from the main body signal under the assumption that the amplitude of the main body signal is invariable, however, this assumption is not always met in practice leading to its performance degeneration. In [14], a method based on sparse representation using multiple sparse Bayesian learning (MSBL) was introduced to preserve the main body signal whereas the micromotion scatterer signal was suppressed. In [15], the joint sparsity feature of the main body signal in the spectrogram was exploited, and the markov chain Monte Carlo (MCMC) sampling in the Bayesian inference was utilized to capture this feature. In this way, the main body spectrogram was estimated from the approximate posterior, and a clear image of the main body can be realized by cross-range compression without the interference of the Micro-Doppler signal. However, MCMC sampling suffers from a heavy computational burden.

For a micromotion scatterer with a small rotating radius which is less than half of the range resolution, the Micro-Doppler signal has the same straight-line shape as the main body signal in the spectrogram. Therefore, the methods mentioned above become invalid in this circumstance. Some methods based on sparse time-frequency representation (STFR) have been developed to address the problem of the Micro-Doppler signal removal in this situation. L.Stankovic et al. proposed a method based on L-statistics which performs the short-time Fourier transform (STFT) to the echo in the contaminated range cell and applies L-statistics estimation to the STFT entries to remove the micromotion signal [18]. However, the main drawback is that it may bring about a high sidelobe level in the imaging result. In [19], a method based on histogram analysis was proposed to remove the Micro-Doppler signal. In [20], the joint sparsity of frequency representation of the main body signal was exploited and a novel method under the sparse representation framework was developed to preserve the components of the main body signal whereas the interference of the micromotion counterparts in time-frequency domain was eliminated. Besides these efforts, another line of works based on the empirical mode decomposition (EMD) and its variants concentrate on the problem of Micro-Doppler separation, but these methods lack theoretical analysis [21,22,23,24].

Low-rank matrix recovery (LRMC) theory [25,26], is a new signal processing method which is proposed in the framework of CS theory. LRMC has attracted a lot of attention over the past few years and has been explored for a wide range of applications, such as medical imaging [27], hyper-spectral imaging (HIS) [28], synthetic aperture imaging (SAR) [29], and digital image processing, etc. The basic idea behind this theory is to recover a matrix that is the sum of a low-rank matrix L and and a sparse matrix S from a small set of linear measurements of the form Y=A(L+S), where A involves a linear operator. This model subsumes three important classes of signal recovery problems: CS, affine rank minimization, and RPCA. The availability of RPCA has been examined, and a variety of convex relaxation methods have been proposed to solve this problem.

In this paper, we establish a new optimization problem for ISAR imaging for a target with micromotion parts, especially for rotating parts, under the framework of RPCA theory. This work is inspired by the inherent outstanding performance of PRCA theory. To be specific, we combine the sparsity of ISAR image and the low-rank property of the matrix associated with Micro-Doppler interference in the range-doppler (RD) domain, which will be investigated and verified by using singular value decomposition singular-value decomposition (SVD) [30] method in the following section. The presented problem involves two tasks, one is ISAR imaging of the main body and the other is Micro-Doppler signal separation. To figure out the multitask problem, we adopt the idea of a traditional alternate minimum (AM) algorithm, which solves the two subproblems alternately, to develop an efficient numerical algorithm. In this way, the solution to the original problem is decomposed into two individual subproblem. To be specific, in the stage of ISAR imaging, the signal components associated with the micromotion scatterers are taken away from the sampled echo data. Meanwhile, the signal components related to the main body are subtracted from the sampled echo data in the stage of Micro-Doppler signal separation as well. We argue that these manipulations may promote the sparsity of ISAR image and enhance the low-rank trait of the matrix associated with Micro-Doppler signal, respectively, in the process of iteration. Each relevant subproblem is solved under the ADMM [31,32], framework. Furthermore, an SVD-free algorithm, which only twice matrix inversion operations are needed whereas SVD computation is no longer required, is developed to cope with the subproblem associated with the stage of Micro-Doppler signal separation. Compared with the SVD-aided method which requires time-consuming SVD computation, this approach has the superiority in promoting computational efficiency because it avoids SVD computation. Owing to the sparsity of ISAR images incorporated with the low-rank character of Micro-Doppler signal matrix, the interference of the micromotion counterparts is eliminated to the utmost extent, and the clear ISAR image of the target main body is yielded.

The rest of this paper is organized as follows. Section 2 introduces the signal mode of ISAR imaging for micromotion targets and presents the formulated optimization problem for it. Section 3 provides two proposed Algorithms. Section 4 evaluates the effectiveness of the proposed methods by experiments on simulated data. Finally, conclusions are drawn in Section 5.

## 2. Signal Model and Problem Formulation

Without loss of generality, we focus on the imaging model of micromotion target on the 2D imaging plane, the subsequent discussions are based on the following assumptions.

1. Point-scattering model can be satisfied, i.e., the radar echo is assumed to be a sum of dominant scatterers.
2. The radar echo satisfies the stop–go assumption, i.e., the target is assumed to be static during one pulse duration.
3. The 2-D imaging plane is unchanged in CPI.
4. The translational motion is compensated completely, thus, the target is equivalent to rotate around the image center, which indicates that the target can be stated as a turntable model.
5. The change of aspect angle of the target is so small that the instantaneous range can be approximated by its first-order Taylor expansion.
6. The range migration among the scatterers is so small that it can be ignored in CPI.

### 2.1. Signal Model

**Figure 1:**
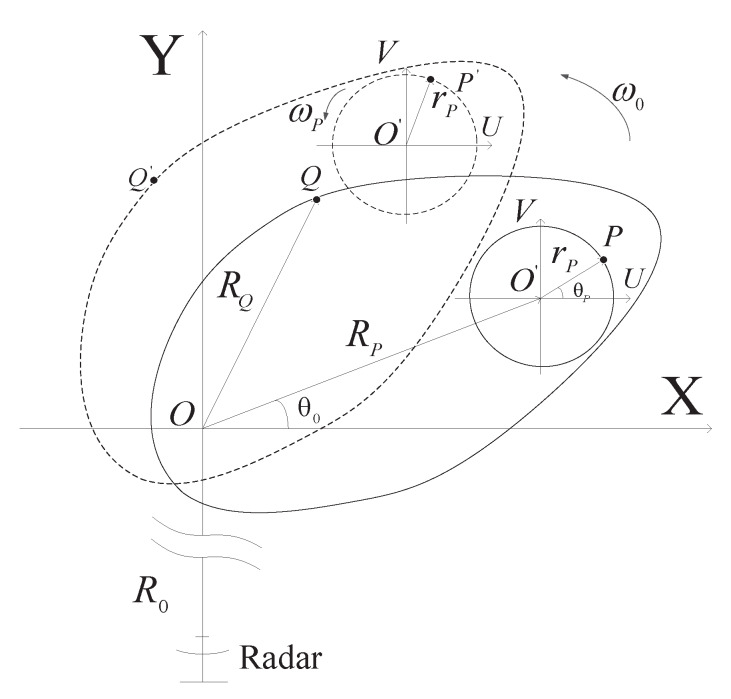
The inverse synthetic aperture radar (ISAR) imaging geometry for a micromotion target.

Figure 1 shows the ISAR imaging geometry for micromotion target. XOY is the imaging plane, scatterer from main body $P(x_{Q},y_{Q})$ rotates uniformly around imaging center O with the radius $R_{Q}$, angular velocity $\omega_{0}$ and the initial angle $\theta_{0}$. Scatterer from rotating part $Q(x_{P},y_{P})$ rotates around $O^{\prime }$ with radius $r_{P}$, angular frequency $\omega_{P}$ and the initial angle $\theta_{P}$. The dotted lines show the change of imaging geometry after a small angle rotation of the main body.

To obtain high range-resolution, ISAR imaging system usually transmits linear frequency-modulated (LFM) waveform. After the preprocessing of demodulation and range compression, the radar echo of a scatterer can be represented as

(1) $$\begin{array}{cc} s\left(t_{m},t_{r}\right) & =\sigma \;\cdot \;sinc\left(B\left(t_{r}-\frac{2R_{\Delta }\left(t_{m}\right)}{c}\right)\right)\\ & \;\cdot \;exp\left(-j\frac{4\pi f_{c}}{c}R_{\Delta }\left(t_{m}\right)\right), \end{array}$$

where *B*, $f_{c}$ and *c* denote the bandwidth, carrier frequency, and light speed, respectively, $t_{m}$ and $t_{r}$ denote the slow and fast time, respectively, σ and $R_{\Delta }\left(t_{m}\right)$ represent the reflection coefficient and instantaneous range between the scatterer and the radar at the slow time $t_{m}$. sinc(x)=sin(πx)/(πx). From Figure 1, we find that the distance between main body scatterer *Q* and reference point *O* satisfies

(2) $$R_{\Delta Q}\left(t_{m}\right)=R_{Q}\sin\left(\omega_{0}t_{m}+\theta_{0}\right)$$

Following the fact that the change of the aspect angle of main body covering in short CPI is small, the instantaneous range $R_{\Delta Q}\left(t_{m}\right)$ can be approximated by its first-order Taylor expansion as

(3) $$\begin{array}{cc} R_{\Delta Q}\left(t_{m}\right) & =R_{Q}+x_{Q}\sin\left(\omega_{0}t_{m}\right)+y_{Q}\cos\left(\omega_{0}t_{m}\right)\\ & =R_{Q}+x_{Q}\omega_{0}t_{m}+y_{Q} \end{array}$$

According to (3) and ignoring the initial phase $\theta_{Q}$, the Doppler frequency of the scatterer Q from main body can be given by

(4) $$f_{dQ}\approx \frac{2\omega_{0}}{\lambda }x_{Q}$$

where $\lambda =c/f_{c}$, (4) suggests the scatterer from main body possesses constant Doppler information approximately.

It can be seen from Figure 1 that $r_{P}\ll R_{0}$ and $R_{P}\ll R_{0}$, then the distance between micromotion scatterer P and reference point O satisfies

(5) $$R_{\Delta P}\left(t_{m}\right)=R_{P}\sin\left(\omega_{0}t_{m}+\theta_{0}\right)+r_{P}\sin\left(\omega_{P}t_{m}+\theta_{P}\right)$$

According to (5) and ignoring the initial phase $\theta_{P}$, the Doppler frequency of the scatterer P can be described as

(6) $$f_{dP}\approx \frac{2\omega_{0}}{\lambda }x_{P}+\frac{2\omega_{P}}{\lambda }r_{P}\cos\left(\omega_{P}t_{m}\right)$$

Therefore, the Micro-Doppler of a rotating scatterer is depicted as a sinusoidal FM signal. It is worthwhile to note that, $f_{dP}$, which is defined as Micro-Doppler signal, will introduce sideband interference around the main body doppler in RD plane and degrade the quality of main body ISAR image.

Based on the assumption that slant-range migration through resolution cells (MTRC) from main body scatter is so small that it can be neglected, therefore, after range alignment, according to (1), (3) and (5), the radar echoes reflected from the target can be represented as

(7) $$\begin{array}{cc} s(t_{m},t_{r}) & =\sum_{Q=1}^{N_{Q}}\sigma_{Q}\\ & \;\cdot \;sinc\left(B\left(t_{r}-\frac{2y_{Q}}{c}\right)\right)\;\cdot \;exp\left(-j\frac{4\pi f_{c}}{c}x_{Q}\omega_{0}t_{m}\right)\\ & +\sum_{P=1}^{N_{P}}\sigma_{P}\;\cdot \;sinc\left(B\left(t_{r}-\frac{2y_{P}}{c}-r_{P}\sin\left(\omega_{P}t_{m}+\theta_{P}\right)\right)\right)\\ & \times exp\left(-j\frac{4\pi f_{c}}{c}\left(x_{P}\omega_{0}t_{m}+r_{P}\sin\left(\omega_{P}t_{m}+\theta_{P}\right)\right)\right)\\ & +\chi (t_{m},t_{r}) \end{array}$$

where $\sigma_{Q}$ and $\sigma_{P}$ denote the signal amplitudes from the *Q*-th main body and *P*-th rotating scatterer, respectively, $N_{Q}$ and $N_{P}$ denote the number of scatterers from main body and rotating parts, respectively. $\chi (t_{m},t_{r})$ is gaussian noise.

For analysis simplicity, we rewrite (7) as (8) in the following

(8) s(tm,tr)=∑Q=1NQσQ′ · exp−j4πfccxQω0tm+∑P=1NPσP′ · exp(−j4πfcc(xPω0tm+rPsin(ωPtm∑P=1NP+θP)))+χ(tm,tr),

where $\sigma_{Q}^{\prime }$ defined as $\sigma_{Q}^{\prime }=\sigma_{Q}\;\cdot \;sinc\left(B\left(t_{r}-\frac{2y_{Q}}{c}\right)\right)$, and $\sigma_{P}^{\prime }$ defined as $\sigma_{P}^{\prime }=\sigma_{P}\;\cdot \;sinc\left(B\left(t_{r}-\frac{2y_{P}}{c}-r_{P}\sin\left(\omega_{P}t_{m}+\theta_{P}\right)\right)\right)$ are equivalent magnitudes from main body and rotating parts signals, respectively.

We can find that $\sigma_{Q}^{\prime }$ is slow time invariant, while $\sigma_{P}^{\prime }$ can be depicted as a sinusoid in $t_{m}$-$t_{r}$ domain. The discrete form corresponding to (8) can be stated as

(9) $$\begin{array}{cc} s(m,n) & =\sum_{Q=1}^{N_{Q}}\sigma_{Q}^{\prime }\left(m,n\right)\;\cdot \;exp\left(-j\frac{4\pi f_{c}x_{q}\omega_{0}}{c\;\cdot \;\mathrm{PRF}}m\right)\\ & +\sum_{P=1}^{N_{P}}\sigma_{P}^{\prime }\left(m,n\right)\;\cdot \;exp(-j\frac{4\pi f_{c}}{c}(\frac{x_{P}\omega_{0}}{\mathrm{PRF}}m\\ & +r_{P}\sin\left(\frac{x_{P}\omega_{P}}{\mathrm{PRF}}m+\theta_{P}\right)))\\ & +\chi (m,n) \end{array}$$

where PRF is pulse repetition frequency of the radar system, m=0,1,⋯,M−1 and n=0,1,⋯,N−1 denote the indices of the slow and fast time, respectively. *M* and *N* are the number of range and doppler cells of the full aperture data. χ(m,n) is the discrete form of Gaussian noise.

According to (9), the mathematical model for sparse aperture ISAR (SA-ISAR) imaging can therefore be given as the following linear equation:

(10) S=F(X+D)+N

where $S\in C^{L\times N}$, $F\in C^{L\times K}$, $X\in C^{K\times N}$, $D\in C^{K\times N}$ and $N\in C^{L\times N}$ denote the range compressed radar echo with SA, the partial Fourier matrix, the unknown pure ISAR image, the adverse Micro-Doppler interference corresponding to the second term in (9), and the complex Gaussian noise, respectively. *L* and *K* are the number of pulses and the reconstructed doppler frequency cells, respectively. The (l,k)-th element of F is $exp(-j2\pi \frac{lk}{N})$. Our aim is to recover X from the observed data S.

### 2.2. Preliminary

**Figure 2:**
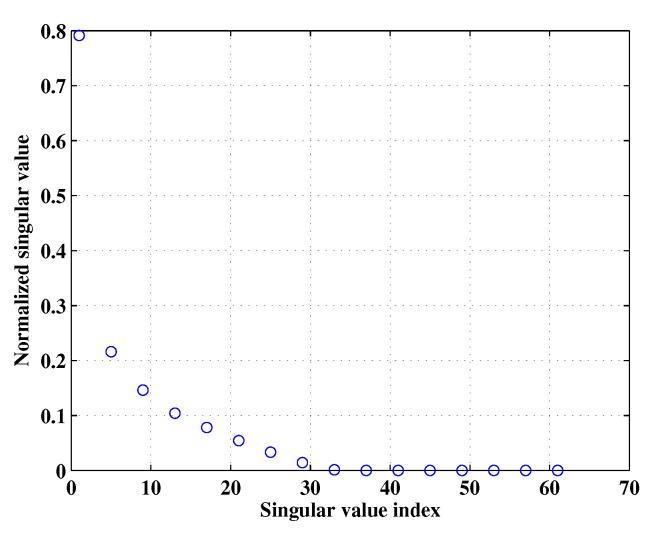
Singular value distribution of simulated ISAR data.

**Figure 3:**
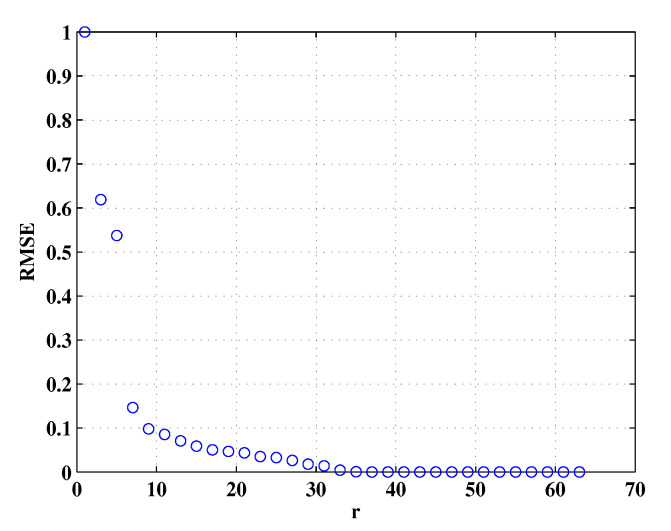
Root Mean Square Error (RMSE) values between the rank-*r* approximated matrix $D_{r}$ and the original matrix D with respect to different values of *r*.

In this subsection, we investigate the property of Micro-Doppler effect in RD domain and reveal the fact that D has a low-rank feature. It is known that the truncated singular value decomposition (TSVD) technique offers the rank-*r* approximation of a given matrix by using the *r*-dominated singular values. Herein, we analyze the low-rank property of D by using TSVD and the details are given below. By applying SVD to a given matrix D, we have $D=U\Lambda V^{H}$, where ${\left(\;\cdot \;\right)}^{H}$ denotes conjugate transposition operator, $U\in C^{K\times K}$ and $V\in C^{N\times N}$ are orthogonal matrices, $\Lambda \in R^{K\times N}$ is is a diagonal matrix with singular values of D. comprising non-negative singular values of D in decreasing order. Then, the approximated matrix of D, denoted as $D_{r}=U_{r}\Lambda_{r}{V_{r}}^{H}$, where $U_{r}=U\left(:,1:r\right)$, $V_{r}=V\left(:,1:r\right)$ and $\Lambda_{r}\in R^{r\times r}$ is a diagonal matrix with its diagonal elements are the *r* largest singular values of D. To quantitatively evaluate the difference between D and $D_{r}$, Root Mean Square Error (RMSE), which is defined as $\mathrm{RMSE}=∥D-D_{r}{∥}_{F}/{∥D∥}_{F}$, is introduced, ${∥\;\cdot \;∥}_{F}$ denotes Frobenius norm. In this simulation, the number of rotating scatterers, the fast and slow time samples were set as 2, 128 and 128, respectively, the received data S were generated according to (9), the normalized singular values of it are shown in Figure 2. We can see that the distribution of the singular values decays fast. The RMSE of D and $D_{r}$ is shown in Figure 3, we can see that, D can be well approximated by a matrix $D_{r}$, meanwhile, when the top 40 largest singular values are employed to compute $D_{r}$, the reconstruction error is very small indicating that $D_{r}$ is capable of capturing the most energy of D. As mentioned above, we argue that both Figure 2 and Figure 3 shed light on the low-rank of D. It is pointed out that the prior knowledge about the Micro-Doppler signal is the basis of our approaches proposed in this paper.

### 2.3. Proposed Optimization Problem

To reconstruct the ISAR image from SA sampled data, prior knowledge about the ISAR image and Micro-Doppler interference are utilized to formulate the ISAR imaging problem in this subsection.

Recalling (10), to restore the ISAR image from the down-sampled echo data with Micro-Doppler interference, we propose the following optimization problem based on RPCA theory:

(P0) $$\begin{array}{c} \left(X^{*},D^{*}\right)=\underset{X,D}{\mathrm{argmin}}\;\mathrm{rank}\left(D\right)+\lambda \;\cdot \;{\left\|X\right\|}_{0}\\ s.t.\;S=F(X+D) \end{array}$$

where ${\left\|X\right\|}_{0}$ denotes $\ell_{0}$ norm. rank denotes rank function and λ>0 is is a regularization parameter.

Unfortunately, P0 is a NP-hard problem due to the existences of rank( · ) function and ${\left\|\;\cdot \;\right\|}_{0}$ norm. Many efforts have been made to resolve this problem, we relax P0 by substituting rank(D) and ${\left\|X\right\|}_{0}$ as ${\left\|D\right\|}_{*}$ and ${\left\|X\right\|}_{1}$ to interpret our work in the rest of this paper. Thus, we have

(P1) $$\begin{array}{c} \left(X^{*},D^{*}\right)=\underset{X,D}{\mathrm{argmin}}{\;\|D\|}_{*}+\lambda \;\cdot \;{\left\|X\right\|}_{1}\\ s.t.\;S=F(X+D) \end{array}$$

where ${\left\|\;\cdot \;\right\|}_{*}$ denotes convex nuclear norm, which is defined as ${\left\|X\right\|}_{*}=\mathrm{trace}\left(\sqrt{X^{H}X}\right)$, herein trace denotes matrix trace. ${\left\|\;\cdot \;\right\|}_{1}$ denotes the convex $\ell_{1}$ norm. Wright et al. [26] proved that the convex relaxation formation P1 can exactly recover the low-rank and sparse matrices under some mild conditions.

## 3. Proposed Algorithms

In this subsection, we design two efficient numerical algorithms to seek a solution for the problem P1.

### 3.1. Algorithm 1

Inspired by the ideal of the AM method, we decompose P1 into two subproblems described as follows

(11) $$\begin{array}{cc} & X^{\left(k+1\right)}=\underset{X}{\mathrm{argmin}}{\left\|X\right\|}_{1}\;\;\\ & s.t.\;\mathrm{FX}=S_{X}\;,\mathrm{wherein}\;\;S_{X}=S-FD^{\left(k\right)} \end{array}$$

(12) $$\begin{array}{cc} & D^{\left(k+1\right)}=\underset{D}{\mathrm{argmin}}{\left\|D\right\|}_{*}\;\\ & s.t.\;\mathrm{FD}=S_{D}\;,\mathrm{wherein}\;S_{D}=S-FX^{\left(k+1\right)} \end{array}$$

It is worthwhile to point out that, as shown in (11) and (12), for the (k+1)-th iteration, we subtract the last estimated $D^{\left(k\right)}$ from S in updating $X^{\left(k+1\right)}$, meanwhile, the updated $X^{\left(k+1\right)}$ is subtracted from S in updating $D^{\left(k+1\right)}$. In this way, the sparsity of X will be promoted and the low-rank of D will be boosted in the current iterative process.

The problem of (11) and (12) can be expressed in the unconstrainted forms as following:

(13) X(k+1)=argminXλx‖X‖1+0.5‖SX−FX‖F2,=wherein SX=S−FD(k)

(14) D(k+1)=argminDλd‖D‖*+0.5‖SD−FD‖F2,=wherein SD=S−FX(k+1)

where $\lambda_{x}$ and $\lambda_{d}$ are regularization parameters.

**Remark** **1.** *The regularization parameter plays an important role in indicating a tradeoff between the data fitting error and the sparsity of the solution. The regularization parameter selection is still an open problem, in [33], the authors presented a two-stage approach to select the regularization parameter which is not discussed further herein for brevity. We apply this method to select the regularization parameter in this paper.*

1. **solution for** (13)
  To solve the optimization problem (13), the ADMM method is employed, and the main procedures are derived in the following. For the sake of simplicity, the superscripts (k) and (k+1) are omitted.
  To apply the ADMM method, introducing an auxiliary variable $\tilde{X}\in C^{K\times N}$ and the Lagrange multiplier $\mathrm{dX}\in C^{K\times N}$ is required. Then we split the variable X as $X=\tilde{X}$, having the augmented Lagrangian function as
  (15) J=maxX˜dXminX,X˜λx‖X˜‖1+0.5‖SX−FX‖F2+0.5β‖X−X˜+dX/β‖F2
  where β>0 is a step size.
  For the problem (15), we alternately solve the following subproblems as
  $$\begin{array}{c} X:=\underset{X}{\mathrm{argmin}}\frac{1}{2}{\left\|S_{X}-\mathrm{FX}\right\|}_{F}^{2} \end{array}$$
  (16) $$\begin{array}{c} +\frac{\beta }{2}{\left\|X-\tilde{X}+\mathrm{dX}/\beta \right\|}_{F}^{2} \end{array}$$
  (17) $$\begin{array}{c} \tilde{X}:=\underset{\tilde{X}}{\mathrm{argmin}}\lambda_{x}\|\tilde{X}{\|}_{1}+\frac{\beta }{2}{\left\|X-\tilde{X}+\mathrm{dX}/\beta \right\|}_{F}^{2} \end{array}$$
  (18) $$\begin{array}{c} \mathrm{dX}:=\mathrm{dX}+\beta (X-\tilde{X}) \end{array}$$
  Problem (16) involves a quadratic cost and leads to a closed-form solution, which can be obtained by setting the first-order derivative of its objective function with respect to X as zero. We obtain
  (19) $$\begin{array}{c} X:={\left(F^{H}F+\beta I\right)}^{-1}\times \left(F^{H}S_{X}+\beta \tilde{X}+\mathrm{dX}\right) \end{array}$$
  Problem (17) has a closed solution involving $\ell_{1}$ norm shrink operator [31]:
  (20) $$\begin{array}{c} \tilde{X}:=shrink\left(X+\mathrm{dX}/\beta ,\lambda_{x}/\beta \right) \end{array}$$
  where $shrink\left(x,\zeta \right)=sign\left(x\right).*\max\left(|x|-\zeta \right)$.
2. **solution for** (14)
  For the problem (14), the splitting variable $D=\tilde{D}\in C^{K\times N}$ and the Lagrange multiplier $\mathrm{dD}\in C^{K\times N}$ are required. Then we have the augmented Lagrangian function as:
  (21) J=maxD˜dDminD,D˜λd‖D˜‖*+0.5‖SD−FD‖F2 +0.5τ‖D−D˜+dD/τ‖F2
  where τ>0 is a stepsize.
  For problem (21), we alternately solve the following subproblems:
  $$\begin{array}{c} D:=\underset{D}{\mathrm{argmin}}\frac{1}{2}{\left\|S_{D}-\mathrm{FD}\right\|}_{F}^{2} \end{array}$$
  (22) $$\begin{array}{c} +\frac{\tau }{2}{\left\|D-\tilde{D}+\mathrm{dD}/\tau \right\|}_{F}^{2} \end{array}$$
  (23) $$\begin{array}{c} \tilde{D}:=\underset{\tilde{D}}{\mathrm{argmin}}\lambda_{d}\|\tilde{D}{\|}_{*}+\frac{\tau }{2}{\left\|D-\tilde{D}+\mathrm{dD}/\tau \right\|}_{F}^{2} \end{array}$$
  (24) $$\begin{array}{c} \mathrm{dD}:\;=\mathrm{dD}+\tau (D-\tilde{D}) \end{array}$$
  Problem (22) has a closed solution, which is represented as
  (25) $$\begin{array}{c} D:={\left(F^{H}F+\tau I\right)}^{-1}\times \left(F^{H}S_{D}+\tau \tilde{D}+\mathrm{dD}\right) \end{array}$$
  The problem (23) involves a nuclear norm minimization problem, which can be solved by SVT computation in [30]:
  (26) $$\begin{array}{c} \tilde{D}:=svt\left(D+\mathrm{dD}/\tau ,\lambda_{d}/\tau \right) \end{array}$$
  where $svt\left(X,\zeta \right)=U\;\cdot \;diag\left([\max(\zeta ,0)]\right)\;\cdot \;V^{H}$, wherein $X=U\;\cdot \;diag\left(\zeta \right)\;\cdot \;V^{H}$ is the SVD of X.
  The whole algorithm is summarized in Algorithm 1 (Micro-doppler Extraction based on RPCA).

**Algorithm 1** ME-RPCA.
1:*Input:*S, F, $\lambda_{x}$, $\lambda_{d}$, β, τ.  2:*Initialization:*  $X^{\left(0\right)}=F^{H}S$, ${\tilde{X}}^{\left(0\right)}={\mathrm{dX}}^{\left(0\right)}=0_{K\times N}$, $D^{\left(0\right)}={\tilde{D}}^{\left(0\right)}$, k=0.      % outer iteration  3:**while** not converged **do**4:  
$S_{X}=S-FD^{\left(k\right)}$   % inner 1 iteration5:  **while** not converged **do**6:   update $X^{\left(k+1\right)}$ using (19); 7:   update ${\tilde{X}}^{\left(k+1\right)}$ using (20); 8:   update ${\mathrm{dX}}^{\left(k+1\right)}$ using (18);  9:  **end while**  10:  $S_{D}=S-FX^{\left(k+1\right)}$ % inner 2 iteration  11:  **while** not converged **do**12:   update $D^{\left(k+1\right)}$ using (25); 13:   update ${\tilde{D}}^{\left(k+1\right)}$ using (26); 14:   update ${\mathrm{dD}}^{\left(k+1\right)}$ using (24);  15:  **end while**  16:  k = k+1.  17:**end while** 18:*Output:*X. 


### 3.2. Algorithm 2

From (26), it can be seen that, to solve the subproblem (14), a SVD computation is required for the svt operator in each iteration. However, it is time and memory-consuming to perform SVD on a large-scale matrix. To address this issue, N. Srebro has demonstrated that the following relationship holds true [34]:

(27) $$\begin{array}{c} {\left\|D\right\|}_{*}=\min_{U,V}\frac{1}{2}\left({\left\|U\right\|}_{F}^{2}+{\left\|V\right\|}_{F}^{2}\right)\\ s.t.\;D=UV^{H} \end{array}$$

where $U\in C^{K\times d}$, $V\in N^{K\times d}$, and usually d≤min(K,N). With this proxy, we replace ${\left\|D\right\|}_{*}$ with (27) in (14), having

(28) D,U,V=argminD,U,V 0.5λd‖U‖F2+‖V‖F2D,U,V+0.5‖SD−FD‖F2s.t.  D=UVH

3. **solution for** (28).
  The augmented Lagrangian form of (28), after simple mathematic manipulation, is
  (29) J=argminD,U,Vλd‖U‖F2+‖V‖F2+‖SD−FD‖F2 J+γ ‖D−UVH+D˜/γ‖F2
  where $\tilde{D}$ denotes Lagrange multiplier, γ>0 is the stepsize.
  According to (29), the resulting ADMM steps are expressed as follows:
  $$\begin{array}{c} D:=\underset{D}{\mathrm{argmin}}{\left\|S_{D}-\mathrm{FD}\right\|}_{F}^{2} \end{array}$$
  (30) $$\begin{array}{c} +\gamma \;\|D-UV^{H}+\tilde{D}/\gamma {\|}_{F}^{2} \end{array}$$
  (31) $$\begin{array}{c} U:=\underset{U}{\mathrm{argmin}}\lambda_{d}{\left\|U\right\|}_{F}^{2}+\gamma {\left\|D-UV^{H}+\tilde{D}/\gamma \;\right\|}_{F}^{2} \end{array}$$
  (32) $$\begin{array}{c} V:=\underset{V}{\mathrm{argmin}}\lambda_{d}{\left\|V\right\|}_{F}^{2}+\gamma {\;\|D-UV^{H}+\tilde{D}/\gamma \|}_{F}^{2} \end{array}$$
  (33) $$\begin{array}{c} \tilde{D}:=\tilde{D}+\tau_{1}\left(D-UV^{H}\right) \end{array}$$
  where $\tau_{1}>0$ is a stepsize.
  Obviously, all of the problems associated with (30), (31) and (32) are least squares problems, so that their optimal solutions can be obtained by setting the first-order derivative of corresponding objective functions with respect to the target variables. After some manipulations we have
  (34) $$\begin{array}{c} D:={\left(F^{H}F+\gamma I\right)}^{-1}\left(F^{H}S_{D}+\gamma UV^{H}-\tilde{D}\right) \end{array}$$
  (35) $$\begin{array}{c} U:=\left(\tilde{D}+\gamma D\right)V{\left(\lambda_{d}I+\gamma VV^{H}\right)}^{-1} \end{array}$$
  (36) $$\begin{array}{c} V:={\left(\tilde{D}+\gamma D\right)}^{H}U{\left(\lambda_{d}I+\gamma U^{H}U\right)}^{-1} \end{array}$$
  The whole algorithm is summarized in Algorithm 2 (Micro-doppler Extraction based on Low Complexity RPCA).

**Algorithm 2** ME-LCRPCA.
1:*Input:*S, F, $\lambda_{x}$, $\lambda_{d}$, γ, $\tau_{1}$, *d*.  2:*Initialization:*  $X^{\left(0\right)}=F^{H}S$, ${\tilde{X}}^{\left(0\right)}={\mathrm{dX}}^{\left(0\right)}=0_{K\times N}$, $D^{\left(0\right)}={\tilde{D}}^{\left(0\right)}$, $U^{\left(0\right)}=rand\left(K,d\right)$, $V^{\left(0\right)}=rand\left(K,d\right)$,   where rand denotes random number, k=0.    % outer iteration 3:**while** not converged **do**4:  $S_{X}=S-FD^{\left(k\right)}$ % inner 1 iteration 5:  **while** not converged **do**6:   update $X^{\left(k+1\right)}$ using (19); 7:   update ${\tilde{X}}^{\left(k+1\right)}$ using (20); 8:   update ${\mathrm{dX}}^{\left(k+1\right)}$ using (18); 9:  **end while** 10:  $S_{D}=S-FX^{\left(k+1\right)}$  % inner 2 iteration 11:  **while** not converged **do**12:   update $D^{\left(k+1\right)}$ using (34); 13:   update $U^{\left(k+1\right)}$ using (35); 14:   update $V^{\left(k+1\right)}$ using (36);15:   update ${\tilde{D}}^{\left(k+1\right)}$ using (33); 16:  **end while** 17:  k = k+1.  18:**end while** 19:*Output:*X. 

**Remark** **2.** *We have carried out many experiments and the simulation results show that the proposed Algorithm 2 converges and can achieve satisfying performance when d is selected as 2–8.*

### 3.3. Convergence Analysis

We examined the convergence behavior and computational complexity of our proposed methods. It has been proved that the ADMM approach endows outstanding performance for solving convex problems with linear equation constraints, and the converge is guaranteed under mild conditions. From the previous analysis, our proposed algorithms are formulated within the AM framework in which the ADMM approach is incorporated to solve the two relevant convex subproblems, so the convergence of our algorithms is guaranteed.

We compare their computation complexity. For Algorithm 1, a SVD calculating is required to update $\tilde{D}$ in (26), which occupies the largest complexity cost, $o\left(\min(K,N)\times K\times N\right)$. For Algorithm 2, calculating (35) and (36) is needed to update D, which involves twice matrix inversion, having a time cost $2\times o(d^{3})$. Recalling that *d* herein is a very small number, so that the costs of calculating (35) and (36) are extremely low. We make a statement with confidence that the Algorithm 2 is superior to Algorithm 1 when considering the computation cost.

## 4. Experiments

In this section, several experiments based on simulated data were carried out to demonstrate the effectiveness of the proposed algorithms. Moreover, all the experiments are coded by Matlab (version 2014a) and run on a PC with Intel(R) Core (TM) 3.1GHz i7 CPU and 8.0 GB RAM. In addition, for both Algorithms 1 and 2, they stop when the outer iteration, inner 1 iteration, and inner 2 iterations satisfy the following criterions $\|X^{k+1}-X^{k}{\|}_{F}^{2}/{\left\|X^{k}\right\|}_{F}^{2}<10^{-4}$, $\|X^{k+1}-X^{k}{\|}_{F}^{2}/{\left\|X^{k}\right\|}_{F}^{2}<10^{-4}$ and $\|D^{k+1}-D^{k}{\|}_{F}^{2}/{\left\|D^{k}\right\|}_{F}^{2}<10^{-4}$, respectively, and the maximum iteration number of outer, inner 1 and 2 iterations are set as 160, 5 and 5, respectively.

**Figure 4:**
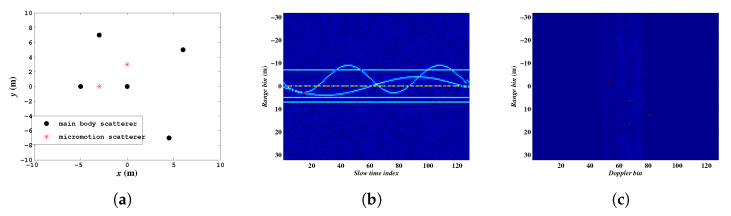
(**a**) The scatterer model of the target; (**b**) The high-resolution range profile (HRRP) sequence of the full data after pulse compression; (**c**) Imaging result of range-doppler algorithm (RDA) with the full data.

The target main body embraces five scatterers, and besides those, it has two rotating scatterers. The translational motion is assumed to be compensated completely, and only the rotational motion of the main body with a rate of 0.02 rad/s is preserved. The two rotating scatterers rotate around the origin with a radius of 6 and 4 m, and with rotating frequencies of 10 and 5 Hz, respectively. It is assumed that the radar system uses X-band, and the center frequency, bandwidth, PRF, and pulse width are 20 GHz, 0.3 GHz, 100 Hz, and 100 μs, respectively. The full data are composed of 128 azimuth samples, and each of them contains 128 fast time samples. Figure 4a shows the scatterer model of the target, Figure 4b shows the high-resolution range profile (HRRP) sequence of the full data after pulse compression, and Figure 4c gives the imaging result of RDA with the full data which were used as a reference for performance comparisons. Besides, to quantitatively evaluate the performance of the proposed algorithms, the entropy of the recovered image *p*, which is defined as $\mathrm{entropy}=-\sum_{i}p\left(i\right)\log p\left(i\right)$, is adopted.

**Figure 5:**
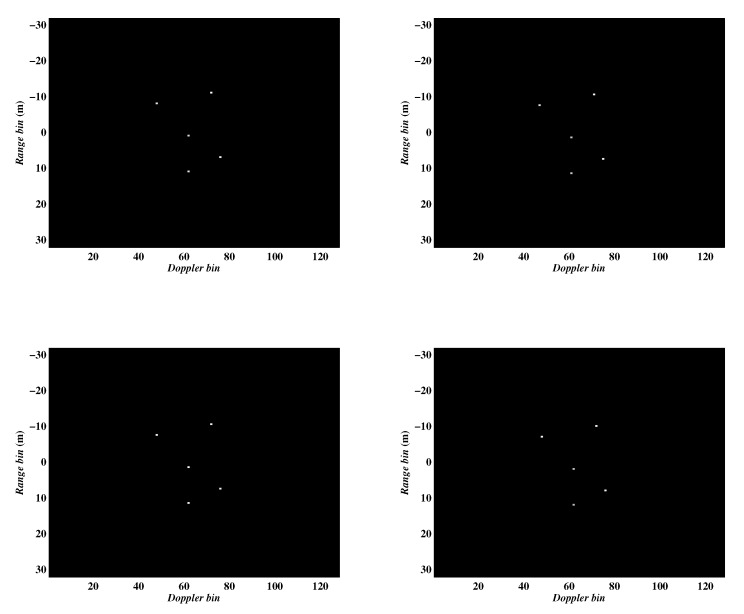
ISAR images obtained by the two proposed algorithms under different sampling schemes. Column 1: continuously sampling. Column 2: random sampling. Row 1: Algorithm 1. Row 2: Algorithm 2.

In the first experiment, we examined the performance of the proposed algorithms with different sampling schemes, namely continuously and randomly sampling schemes. The parameters for this experiment were set as: the complex Gaussian noise was added to each pulse to simulate the noise environment with signal-to-noise ratio (SNR) (, which is defined as $\mathrm{SNR}(\mathrm{dB})=10\log_{10}\left(\frac{P_{signal}}{P_{noise}}\right)$, where $P_{signal}$ and $P_{noise}$ are power of signal and noise, respectively,) equal to 10 dB, the number of pulses was set to 64. The imaging results were shown in Figure 5. The first column of the figures was generated by selecting the first 64 pulses of the full data, and the second column of figures was generated by randomly selecting 64 pulses from the full data. Meanwhile, the first and second rows present the images obtained by Algorithms 1 and 2, respectively. It can be seen that the two methods can achieve rather similar clear images confirming that both of them are capable of removing Micro-Doppler interference excellently. The entropies of the imaging results corresponding to Figure 5 and CPU running times are listed in Table 1. We can see that the computation efficiency of Algorithm 2 is more efficient than Algorithm 1, which demonstrates the conclusion mentioned in the previous section in terms of quantitative analysis.

**Figure 6:**
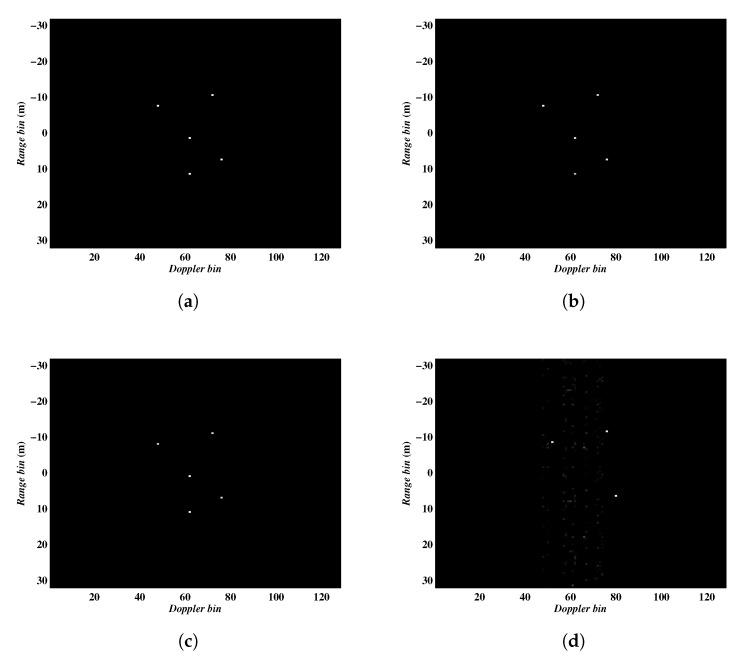
ISAR images obtained by Algorithm 2 using different number of pulses. (**a**–**d**) corresponding to results from 64, 32, 16, 8 sampled pulses, respectively.

Next, we testify the performance of the algorithms in terms of different pulse numbers. It is well-known that the performance of CS relies on the measurement number, i.e., the pulse number in our case. To investigate the role of the pulse number, the parameters for this experiment were set as follows. The complex Gaussian noise was added to each pulse to simulate the noise environment with SNR = 10 dB, and a continuously sampling scheme was employed to select pulses from the full data. In this simulation, we only show the imaging results obtained by the Algorithm 2, and the results acquired by the Algorithm 1 are almost the same. The ISAR imaging results obtained with a different number of pulses were shown in Figure 6, in which the first, second, third and fourth rows present the results from 64, 32, 16, 8 sampled pulses, respectively. It can be seen that the Algorithm 2 can achieve satisfying imaging results even in the case of 12.5% sampling ratio. In other words, it is more tolerant of data deficiency with a small amount of sampled data. Additionally, the entropies of the figures in Figure 6 were given in Table 2.

**Figure 7:**
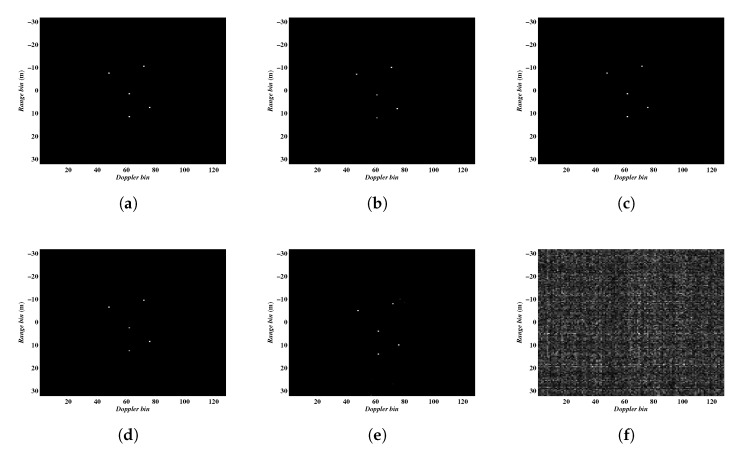
ISAR imaging results obtained under different SNRs. (**a**–**f**) corresponding to the results under 10, 5, 0, −5, −10 and −15 dB, respectively.

We now testify the performance of the methods in terms of different SNRs. To demonstrate the robustness with respect to noise, the parameters for this experiment were set as follows. The complex Gaussian noise was added to each pulse to simulate the noise environment, 64 pulses continuously sampled from the full data were employed. In this simulation, we only show the imaging results obtained by Algorithm 2, and the results acquired by Algorithm 1 are almost the same. The ISAR imaging results under different noise conditions are shown in Figure 7, in which Figure 7a–f present the results with 10, 5, 0, −5, −10 and −15 dB, respectively. The entropies of the imaging results are given in Table 3. It can be seen that Algorithm 2 can achieve satisfying imaging results under low SNR conditions even at −10 dB.

**Figure 8:**
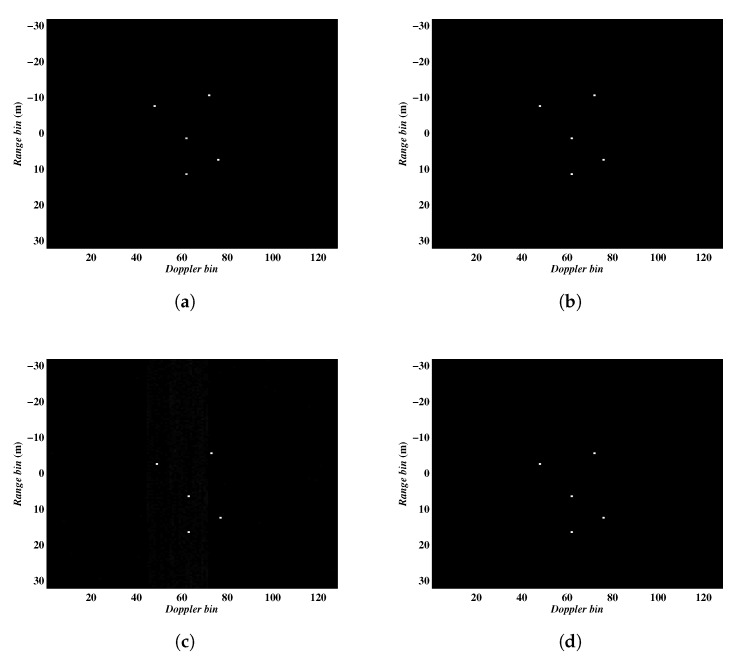
ISAR imaging results obtained by different methods. (**a**–**d**) corresponding to the imaging results by Algorithm 1, Algorithm 2, methods proposed in [14,15], respectively.

In the last experiment, we compare the performance of the proposed algorithms with the previous methods reported in [14,15], which have shown certain excellent performance in removing the Micro-Doppler effect. The parameters for this experiment were set as: the complex Gaussian noise was added to each pulse to simulate the noise environment with SNR = 10 dB, 64 pulses continuously sampled from the full data were employed. The imaging results are shown in Figure 8, in which Figure 8a–d present the results from Algorithm 1, Algorithm 2, methods proposed in [14,15], respectively. In addition, the entropies of the imaging results are given in Table 4.

It can be seen from Figure 8 and Table 4 that our proposed algorithms achieve the minimum entropies, suggesting the performance advantages over the methods proposed in [14,15]. As a matter of fact, although the MSBL method in [14] suppresses the Micro-Doppler signal to some degree, there are residuals leading to image blur. The shortcoming of the method in [15] is that its performance is susceptible to noise, furthermore, it suffers from heavy computing load due to its complicated Monte Carlo sampling process.

## 5. Conclusions

Under the framework of RPCA, two ISAR imaging algorithms for micromotion targets with rotating parts were proposed in this paper. Robust imaging performance can be achieved by the proposed algorithms, even in the low SNR case with grossly inadequate measurements. Furthermore, by resorting to the proxy of the nuclear norm, the proposed SVD-free algorithm avoids computing SVD of the given matrix which improves the computing efficiency. However, there are some limitations in them, if the radius of the rotating part is smaller than half of the range resolution, and/or the rigid body part undergoes maneuvering flying, etc. Additionally, if the radar system suffers from non-Gaussian noise, such as speckle and impulse noise, the performance of our methods would incline to degenerate. In the future, we will further validate the effectiveness of the proposed algorithms by real measured data.

## Figures and Tables

**Table 1:** Entropies of the reconstructed images and CPU times of the proposed algorithms.

		Algorithm 1	Algorithm 2
Continuously	**Entropy**	1.61	1.61
sampling	**CPU time**	7.1 s	**4.6 s**
Randomly	**Entropy**	1.61	1.61
sampling	**CPU time**	7.3 s	**4.7 s**

**Table 2:** Entropies of the imaging results.

	Figure 6a	Figure 6b	Figure 6c	Figure 6d
**Entropy**	1.61	1.60	1.53	3.97

**Table 3:** Entropies of the imaging results.

	Figure 7a	Figure 7b	Figure 7c	Figure 7d	Figure 7e	Figure 7f
**Entropy**	1.61	1.65	1.60	1.67	1.59	7.81

**Table 4:** Entropies of the imaging results and CPU time of methods.

	Figure 8a	Figure 8b	Figure 8c	Figure 8d
**Entropy**	**1.61**	1.67	3.78	1.96
**CPU time**	4.5 s	7.2 s	**3.1 s**	27.8 s
